# LncRNA-SNHG1 promotes macrophage M2-like polarization and contributes to breast cancer growth and metastasis

**DOI:** 10.18632/aging.203609

**Published:** 2021-10-07

**Authors:** Shoukai Zong, Wei Dai, Xiangting Guo, Kai Wang

**Affiliations:** 1 Department of Breast Surgery, The People’s Hospital of Rizhao, Rizhao, Shandong Province, China; 2 Department of Breast and Thyroid Surgery, TCM Hospital of Rizhao, Rizhao, Shandong Province, China; 3 Department of Rheumatology and Immunology, The People’s Hospital of Rizhao, Rizhao, Shandong Province, China; 4 Department of Oncology, The People’s Hospital of Rizhao, Rizhao, Shandong Province, China

**Keywords:** breast cancer, macrophage, polarization, lncRNA, tumor associated macrophages

## Abstract

Breast cancer is one of the most common malignant cancers among women. Cancer cells and adjacent cells determine the development of the disease. Tumor associated macrophages (TAMs) are involved in the regulation of different stages of cancer progression. LncRNAs play an important role in tumor growth and metastasis. However, the function of lncRNA in macrophage and tumor cell interaction is poorly described. Here we reported that lncRNA SNHG1 functioned as a modulator of M2 macrophage polarization and regulated tumor growth and angiogenesis. We indicated that knockdown of SNHG1 inhibited M2 macrophage polarization by suppression of STAT6 phosphorylation. SNHG1 silencing significantly alleviated migration of MCF-7 cells and tube formation of Human Umbilical Vein Endothelial Cells (HUVEC). Furthermore, we found that implantation of cell mixture of MCF-7 cells and macrophages promoted tumor growth and angiogenesis. However, knockdown of SNHG1 in macrophages reversed that effect. Collectively, we demonstrated the important role of lncRNA SNHG1 in macrophages and breast cancer cells interaction. We highlight the essential effect of lncRNA in tumor progression and provide a new method for the prevention and treatment of breast tumor metastasis.

## INTRODUCTION

According to the statistics reported in 2018, breast cancer is the most frequently diagnosed cancer among women worldwide and is the number one killer of them as well [1]. Distant metastasis has been proved to be the main reason for the majority of deaths in breast cancer [2]. Tumor microenvironment (TME) formed mainly by complex cellular components and cellular releasing agents which are secreted by tumor cells and adjacent cells, such as cytokines, chemokines, and growth factors, is usually believed to contribute to the metastasis of tumor cells [3, 4]. Thus, it is crucial for us to identify the potential and key molecules or steps in TME which act as promotor to cancer metastasis.

Macrophage, a vital cell type in the immune system, is reported to participate in the progression of various cancers, and there are up to 50% of tumor associated macrophages (TAM) in breast cancer. Just like macrophages in other tissues, TAMs also reserve the characteristics of heterogeneity and plasticity, upon stimulation in the complex TME, they can differentiate into classically activated M1 type and alternatively activated M2 type [5]. M1 macrophages are activated by certain kinds of cytokines, like LPS, IFN-γ and TNF-α, and are usually immune-active, while M2 macrophages can be activated by IL-4 or IL-13, and they can inhibit the immune reaction [6]. Recent study has confirmed that M2 macrophages activated by CD4^+^T derived IL-4 are the main cell type in various tumor tissues including breast cancer and are involved in tumor progression [7, 8]. Although some of the mechanisms had been reported, for example, TAMs derived VEGF and MMP contributes to the tumor angiogenesis and tumor cell migration [9, 10], the crosstalk between TAMs and tumor progression in breast cancer is still intricate and to be explored as the new specific target for breast cancer treatment.

LncRNAs are a group of non-protein-coding RNAs, which are usually comprised of over 200 nucleotides [11]. More and more studies have revealed that lncRNAs play a vital role in physiological activities, such as cell proliferation, differentiation, and cell cycle regulation [12–14]. Therefore, unexpected expression of lncRNA is associated with the pathological reaction in various diseases, including cancer [15, 16]. LncRNA is reported to be involved in tumorigenesis, tumor metastasis and drug resistance as either oncogenetic or tumor suppressive molecular [17]. LncRNA-SNHG1 is a newly discovered molecule whose coding gene is located at 11q12.3. LncRNA-SNHG1 is overexpressed in many tumor tissues such as non-small lung cancer, esophageal cancer, and hepatocellular carcinoma [18–20]. A study has shown that lncRNA-SNHG1 can promote cancer cell proliferation and migration through inhibiting the expression of p53 [20]. LncRNA-SNHG1 is also reported to contribute to cancer progression by targeting miRNAs, such as miR-101-3p and miR-145-5p in non-small cell lung cancer [21, 22]. However, in breast cancer, the expression and function of lncRNA-SNHG1 are still poorly understood.

In our study, we investigated the role of lncRNA-SNHG1 on macrophage polarization during tumor progression. We found that lncRNA-SNHG1 was an important mediator of M2 macrophage polarization and silencing of lncRNA-SNHG1 significantly reduced the number of F4/80^+^CD206^+^ positive macrophages via inhibition of STAT6 phosphorylation. In addition, silencing of lncRNA-SNHG1 in macrophages inhibited the pro-angiogenesis and tumorigenesis effect of M2-like polarized macrophage. We highlight the essential effect of lncRNA in tumor progression and provide a new method for the prevention and treatment of breast tumor metastasis.

## RESULTS

### LncRNA-SNHG1 was involved in the regulation of M2 macrophage polarization

**Figure 1:**
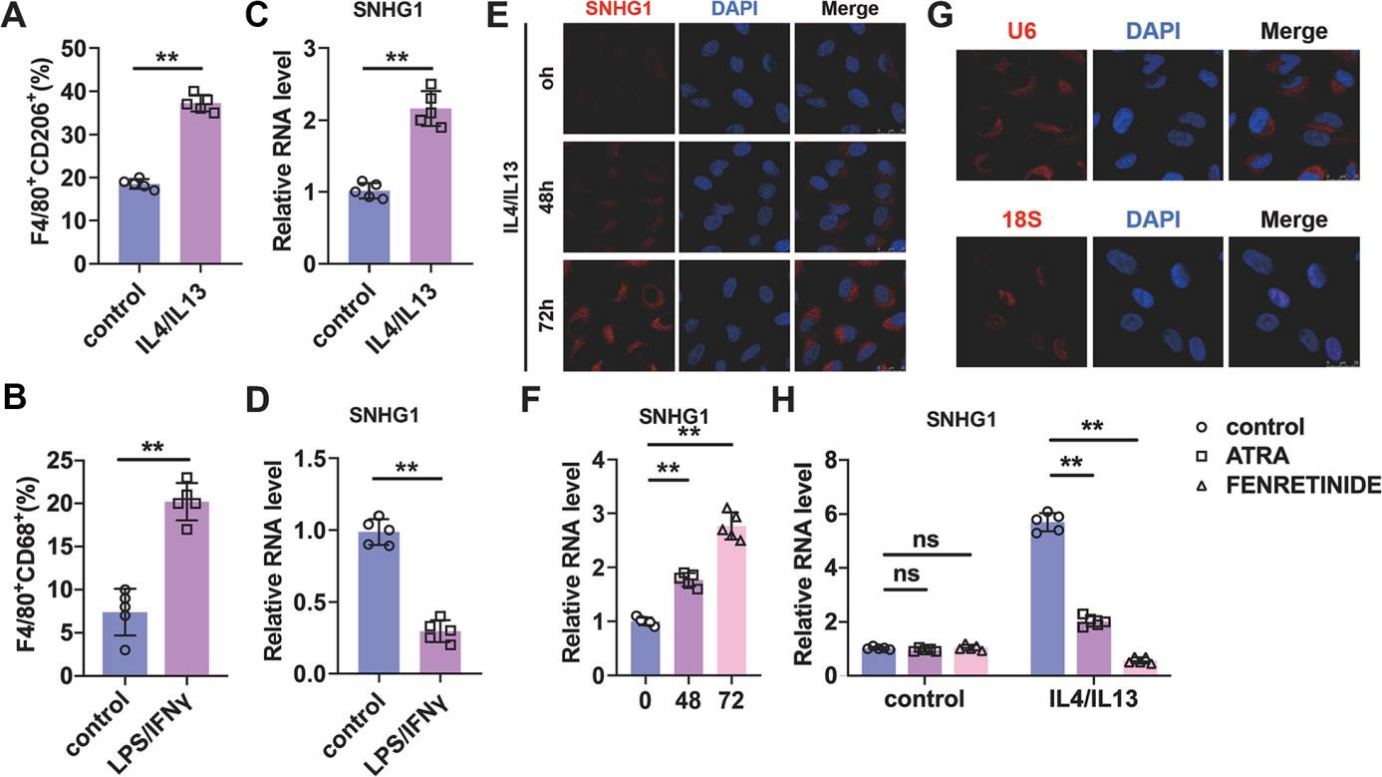
**Identification of lncRNA-SNHG1 as a potential mediator of M2 macrophage polarization.** (**A**, **B**) Flow-cytometric analysis was performed to analyze the percentage of F4/80^+^CD206^+^ or F4/80^+^CD86^+^ cells in BMDMs. (**C**, **D**) qRT-PCR analysis was performed to analyze the RNA level of lncRNA-SNHG1 in BMDMs. BMDMs were treated with IL4/IL13 or LPS/INFγ for 72h. Two-tailed t-test was used for the statistical analysis. n=5 independent cell cultures. The bar indicates the SD values. **P<0.01. (**E**) The FISH assay was conducted to detect the expression of lncRNA-SNHG1. RAW264.7 cells were treated with IL4/IL13 for 72h. (**F**) Quantitative analysis of E. Two-tailed t-test was used for the statistical analysis. n=5 independent cell cultures. The bar indicates the SD values. **P<0.01. (**G**) The localization of U6 snRNA and 18S rRNA was tested by FISH assay in RAW264.7 cells. (**H**) Two inhibitors of M2 macrophage polarization were used to block M2 polarization of RAW264.7 cells. qRT-PCR analysis was performed to analyze the RNA level of lncRNA-SNHG1. Two-tailed t-test was used for the statistical analysis. n=5 independent cell cultures. The bar indicates the SD values. **P<0.01.

To explore the role of lncRNA-SNHG1 in macrophage polarization, we treated bone marrow derived macrophages (BMDMs) with IL4/IL13 (10ng/ml) for 72h to induce BMDMs to M2 subtype and detected the expression level of lncRNA-SNHG1. As shown in Figure 1A, treatment of IL4/IL13 led to an increase of the percentage of F4/80^+^CD206^+^ cells, which indicated the successful induction of M2 macrophage polarization. qRT-PCR analysis was performed to measure the RNA level of lncRNA-SNHG1. We found that IL4/IL13 treatment increased the level of lncRNA-SNHG1 compared to control group and suggested the potential role of lncRNA-SNHG1 in M2 macrophage polarization (Figure 1C). To further explore the role of lncRNA-SNHG1 in macrophage polarization, we treated BMDMs with LPS/IFNγ (10ng/ml) to induce M1 macrophage polarization. Flow-cytometric analysis showed that LPS/IFNγ increased the percentage of F4/80^+^CD68^+^ cells, confirming the induction of M1 macrophage polarization (Figure 1B). Then we detected the RNA level of lncRNA-SNHG1. Compared with control group, treatment of LPS/IFNγ significantly inhibited the expression of lncRNA-SNHG1 (Figure 1D). Furthermore, we conducted probe of lncRNA-SNHG1 to detect the expression and location of lncRNA-SNHG1 via FISH assay. As shown in Figure 1E, 1F, IL4/IL13 promoted the expression of lncRNA-SNHG1 in cytoplasm with a positive correlation with time growth. 18s and U6 were chosen as positive control of nuclear and cytoplasm (Figure 1G). In addition, fenretinide and all-trans retinoic acid (ATRA), two M2 macrophage polarization inhibitors, were used to block macrophage polarization towards M2 subtype. qRT-PCR analysis showed the decrease of lncRNA-SNHG1 in IL4/IL13 treated BMDMs after two inhibitors administration (Figure 1H). Taken together, these data suggested that lncRNA-SNHGl expression was changed in the process of M2 macrophage polarization.

### lncRNA-SNHG1 knockdown attenuated M2 macrophage polarization

**Figure 2:**
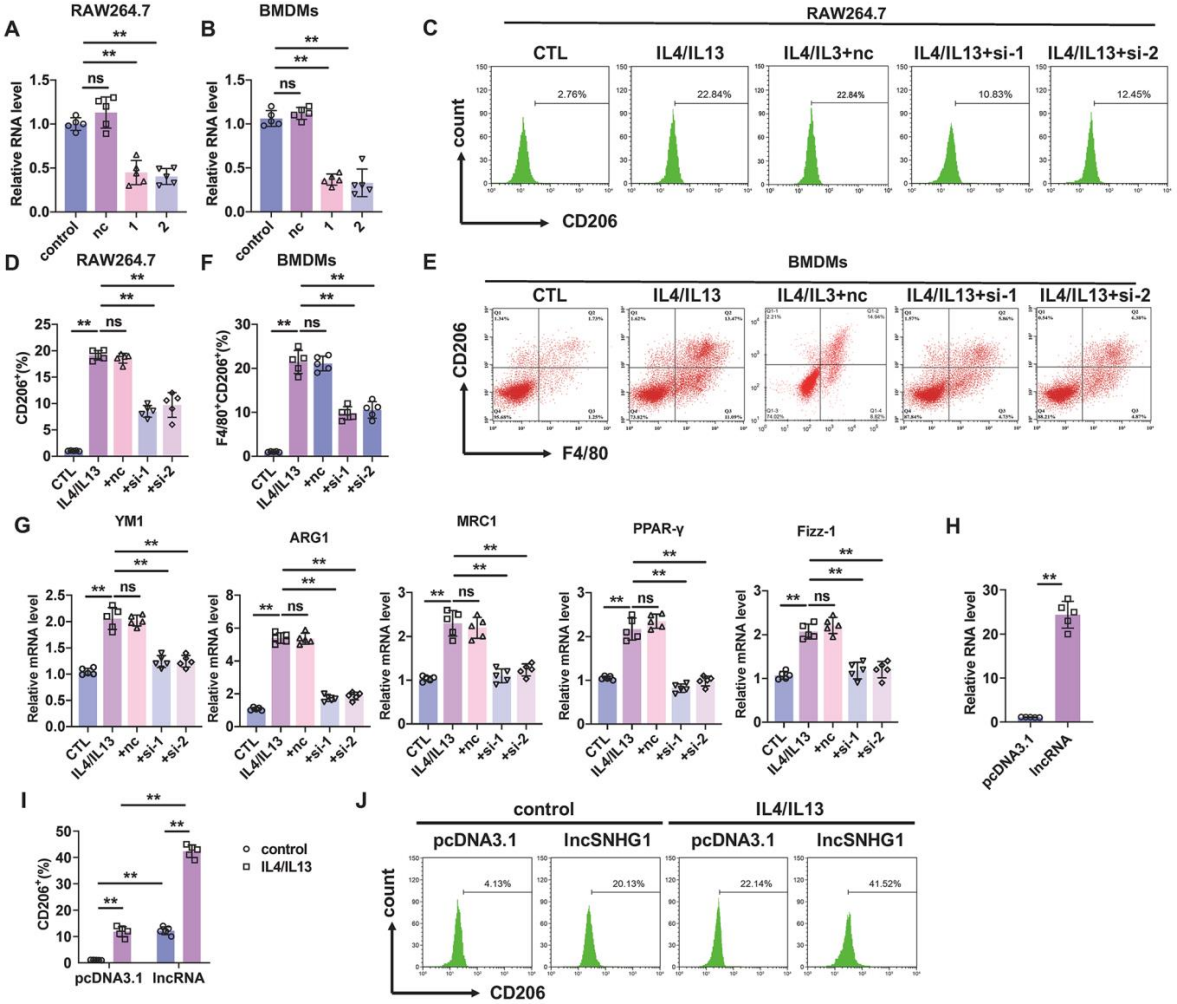
**Silencing of lncRNA-SNHG1 inhibited M2 macrophage polarization.** (**A**, **B**) Two siRNAs of lncRNA-SNHG1 were construct and knockdown efficiency was confirmed in RAW264.7 cells and BMDMs using qRT-PCR analysis. Two-tailed t-test was used for the statistical analysis. n=5 independent cell cultures. The bar indicates the SD values. **P<0.01. (**C**, **D**) Flow-cytometric analysis was performed to analyze the percentage of CD206^+^ cells. RAW264.7 cells were transfected with two siRNAs of lncRNA-SNHG1 and negative control siRNA for 48h and treated with IL4/IL13 or LPS/INFγ for 72h. one-way ANOVA was used for the statistical analysis. n=5 independent cell cultures. The bar indicates the SD values. **P<0.01. (**E**, **F**) Flow-cytometric analysis was performed to analyze the percentage of F4/80^+^CD206^+^ cells. BMDMs were transfected with two siRNAs of lncRNA-SNHG1 or negative control siRNA for 48h and treated with IL4/IL13 or LPS/INFγ for 72h. one-way ANOVA was used for the statistical analysis. n=5 independent cell cultures. The bar indicates the SD values. **P<0.01. (**G**) mRNA of M2 polarization marker genes in RAW264.7 cells was quantified by qRT-PCR analysis. one-way ANOVA was used for the statistical analysis. n=5 independent cell cultures. The bar indicates the SD values. **P<0.01. (**H**) Overexpression of lncRNA-SNHG1 was identified via performing qRT-PCR analysis. pcDNA3.1 empty vector acted as a negative control. one-way ANOVA was used for the statistical analysis. n=5 independent cell cultures. The bar indicates the SD values. **P<0.01. (**I**, **J**) Flow-cytometric analysis was performed to analyze the percentage of CD206^+^ cells. RAW264.7 cells were transfected with expression plasmid of lncRNA-SNHG1 or pcDNA3.1 and treated with IL4/IL13 or LPS/INFγ for 72h. one-way ANOVA was used for the statistical analysis. n=5 independent cell cultures. The bar indicates the SD values. **P<0.01.

We next constructed siRNAs of lncRNA-SNHG1 to inhibit the expression of lncRNA-SNHG1 in BMDMs and macrophage cell line RAW264.7 cells and explored the role of lncRNA-SNHG1 in M2 macrophage polarization. Two siRNAs (si-1 and si-2) of lncRNA-SNHG1 were constructed depend on the RNA sequence of lncRNA-SNHG1. Firstly, qRT-PCR analysis was performed to measure the RNA level of lncRNA-SNHG1 in both RAW264.7 cells and BMDMs, a murine macrophage cell line, after transfection of siRNAs of lncRNA-SNHG1 (Figure 2A, 2B). Compared with the negative control (nc) group, siRNA transfection significantly inhibited lncRNA-SNHG1 level. Then we treated RAW264.7 cells with IL4/IL13 to induce M2 macrophage polarization and transfected them with siRNA of lncRNA-SNHG1 at the same time. We found that inhibition of lncRNA-SNHG1 expression decreased the percentage of CD206^+^ cells (Figure 2C, 2D). Similarly, silencing of lncRNA-SNHG1 also shown an inhibitory effect on M2 macrophage polarization of BMDMs (Figure 2E, 2F). Marker genes of M2 macrophage polarization were also examined to confirm our findings. In line with the results of flow cytometry, mRNA levels of YM1, ARG1, MRC1, PPAR-γ, and Fizz-1 were decreased after lncRNA-SNHG1 knockdown compared with nc group (Figure 2G). Furthermore, we constructed expression plasmid of lncRNA-SNHG1 to overexpress lncRNA-SNHG1 in RAW264.7 cells (Figure 2H). As shown in Figure 2I, 2J, overexpression of lncRNA-SNHG1 increased the percentage of CD206^+^ cells with or without the treatment of IL4/IL13. These data indicated that lncRNA-SNHG1 promoted M2 macrophage polarization.

### Silencing of lncRNA-SNHG1 inhibited the metastasis of tumor cells and angiogenesis

**Figure 3:**
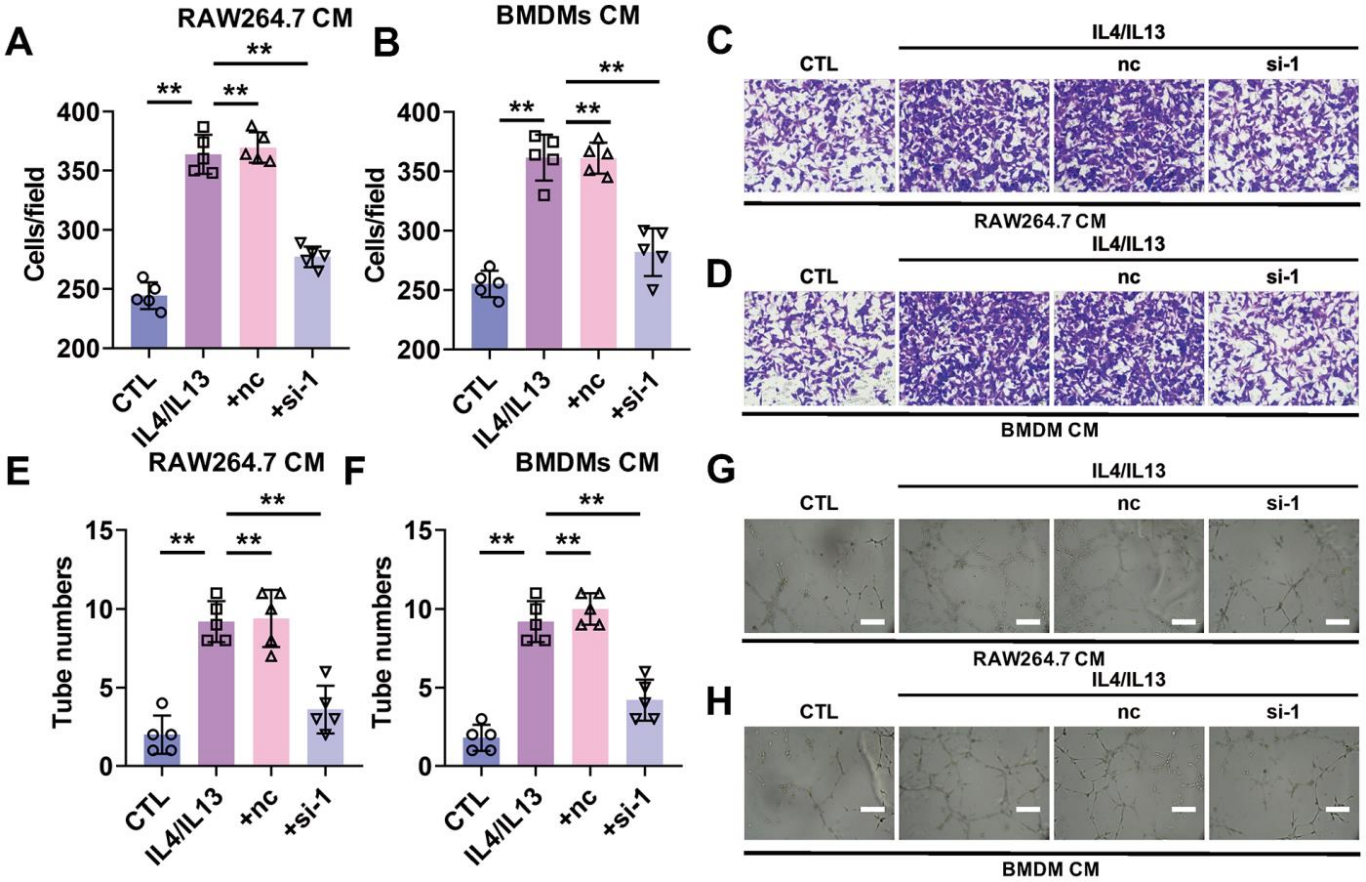
**Silencing of lncRNA-SNHG1 attenuated the pro-angiogenesis effect of M2 macrophage.** MCF-7 cells were cultured using condition medium from RAW264.7 cells or BMDMs for 24 hours. (**A**–**D**) Cell migration of MCF-7 cells cultured with RAW264.7 cells condition medium (**A**, **C**) or BMDMs (**B**, **D**) were assessed by Transwell assay. one-way ANOVA was used for the statistical analysis. n=5 independent cell cultures. The bar indicates the SD values. **P<0.01. (**E**–**H**) Tube formation of HUVEC cells was counted in the presence of the condition medium from RAW264.7 cells (**E**, **G**) or BMDMs (**F**, **H**). one-way ANOVA was used for the statistical analysis. n=5 independent cell cultures. The bar indicates the SD values. **P<0.01.

Then we wanted to know whether silencing of lncRNA-SNHG1 impacted the function of macrophage on tumor development. First, transwell assay was performed to evaluate the effect of macrophage on tumor cell migration after lncRNA-SNHG1 silencing. We chose siRNA-1 of lncRNA-SNHG1 to reduce its expression level. RAW264.7 cells and BMDMs were transfected with siRNA-1 followed by treatment of IL4/IL13 for 48h. Then, condition medium (CM) was changed with medium without serum and collected after 24h. To investigate the influence of lncRNA-SNHG1 on the migration of cancer cells, the serum-free medium collected before was used to culture breast cancer cell line, MCF-7 for 24h. Transwell assay showed that CM from both RAW264.7 cells and BMDMs treated with IL4/IL13 increased the number of migrated MCF-7 cells and decreased after siRNA-1 transfection. (Figure 3A–3D). In addition, we evaluated the role of lncRNA-SNHG1 in angiogenesis. Tube formation experiment indicated that CM from both RAW264.7 cells and BMDMs treated with IL4/IL13 increased the number of luminal formations. However, CM from both RAW264.7 cells and BMDMs transfected with siRNA-1 abolished that effect, decreasing the number of luminal formations (Figure 3E–3H). These data suggested that silencing of lncRNA-SNHG1 attenuated breast cancer cell migration and angiogenesis.

### Silencing of SNHG1 inhibited the phosphorylation of STAT6

**Figure 4:**
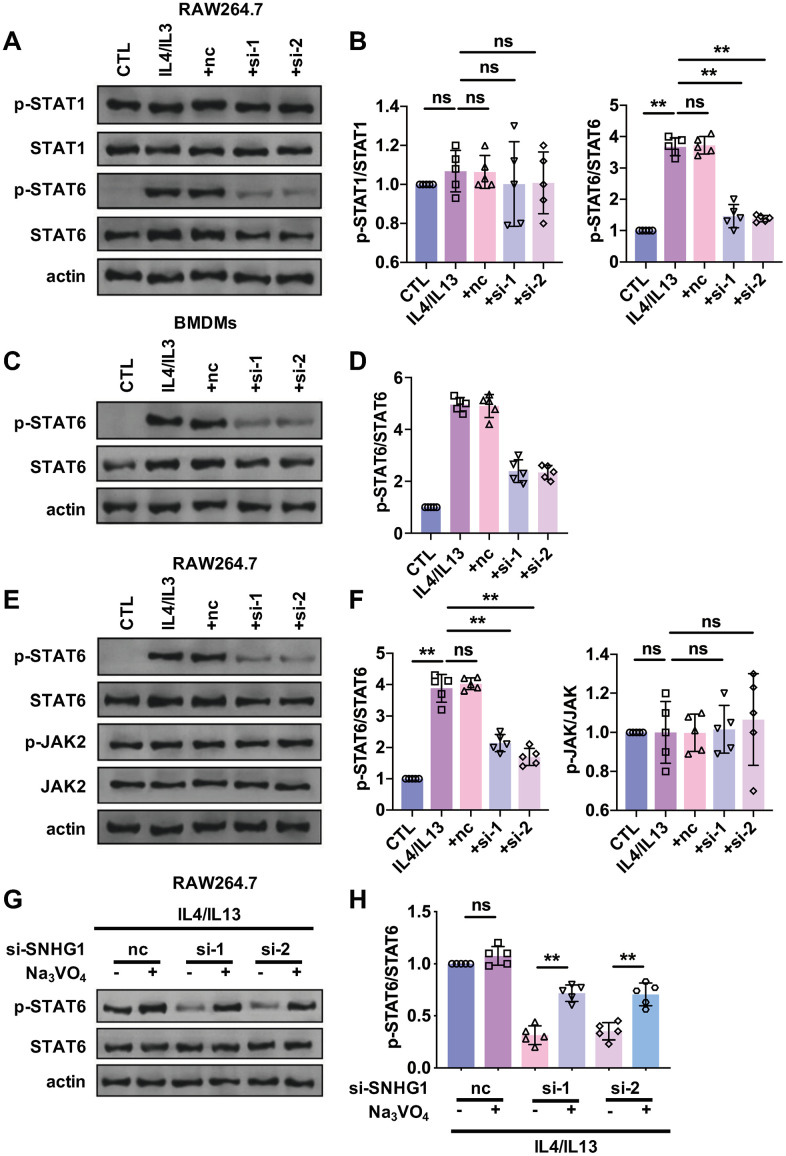
**STAT6 was involved in the regulation of M2 macrophage polarization mediated by lncRNA-SNHG1.** (**A**, **B**) Western blot was performed to detect the protein levels of STAT1, p-STAT1, STAT6, and p-STAT6. RAW264.7 cells were transfected with two siRNAs of lncRNA-SNHG1 and negative control siRNA for 48h and treated with IL4/IL13 or LPS/INFγ for 72h. one-way ANOVA was used for the statistical analysis. n=5 independent cell cultures. The bar indicates the SD values. **P<0.01. (**C**, **D**) Western blot was performed to detect the protein levels of STAT6 and p-STAT6. BMDMs were transfected with two siRNAs of lncRNA-SNHG1 and negative control siRNA for 48h and treated with IL4/IL13 or LPS/INFγ for 72h. one-way ANOVA was used for the statistical analysis. n=5 independent cell cultures. The bar indicates the SD values. **P<0.01. (**E**, **F**) Western blot was performed to detect the protein levels of STAT6, p-STAT6, JAK, and p-JAK. RAW264.7 cells were transfected with two siRNAs of lncRNA-SNHG1 and negative control siRNA for 48h and treated with IL4/IL13 or LPS/INFγ for 72h. one-way ANOVA was used for the statistical analysis. n=5 independent cell cultures. The bar indicates the SD values. **P<0.01. (**G**, **H**) Western blot was performed to detect the protein levels of STAT6 and p-STAT6. RAW264.7 cells were transfected with two siRNAs of lncRNA-SNHG1 and negative control siRNA for 48h and treated with IL4/IL13 or LPS/INFγ for 72h. one-way ANOVA was used for the statistical analysis. n=5 independent cell cultures. The bar indicates the SD values. **P<0.01.

STAT signaling is an important pathway that drives M2 macrophage polarization. To explore whether lncRNA-SNHG1 mediated M2 macrophage polarization via STAT pathway, western blot analysis was performed to detect protein levels of STAT1 and STAT6 and their phosphorylation level. As shown in Figure 4A, 4B, treatment of IL4/IL13 significantly increased the level of p-STAT6, but not p-STAT1 in RAW264.7 cells. And knockdown of lncRNA-SNHG1 by siRNA transfection attenuated that effect and decreasing the phosphorylation level of STAT6. The effect of lncRNA-SNHG1 on phosphorylation of STAT6 was also examined in BMDMs (Figure 4C, 4D). As shown in our FISH data, lncRNA-SNHG1 is localized in cytoplasm. So, we wanted to explore how lncRNA-SNHG1 regulated the phosphorylation of STAT6. JAK2 was reported to mediate the phosphorylation of STAT6. Thus, we measured the protein levels of JAK2 and p-JAK2 by western blot analysis in RAW264.7 cells. We found that IL4/IL13 and lncRNA-SNHG1 silencing had no effect on JAK2 and p-JAK2 levels (Figure 4E, 4F), suggesting that lncRNA-SNHG1 may affect STAT6 phosphorylation by regulating dephosphorylation step. We treated RAW264.7 cells with Na_3_VO_4_, a kind of tyrosine phosphatase inhibitor, and found that Na_3_VO_4_ reversed the inhibitory effect of lncRNA-SNHG1 knockdown on STAT6 phosphorylation (Figure 4G, 4H). These results indicated that lncRNA-SNHG1 regulated phosphorylation of STAT6 to mediated M2 macrophage polarization.

### Knockdown of lncRNA-SNHG1 attenuated tumorigenesis and angiogenesis

**Figure 5:**
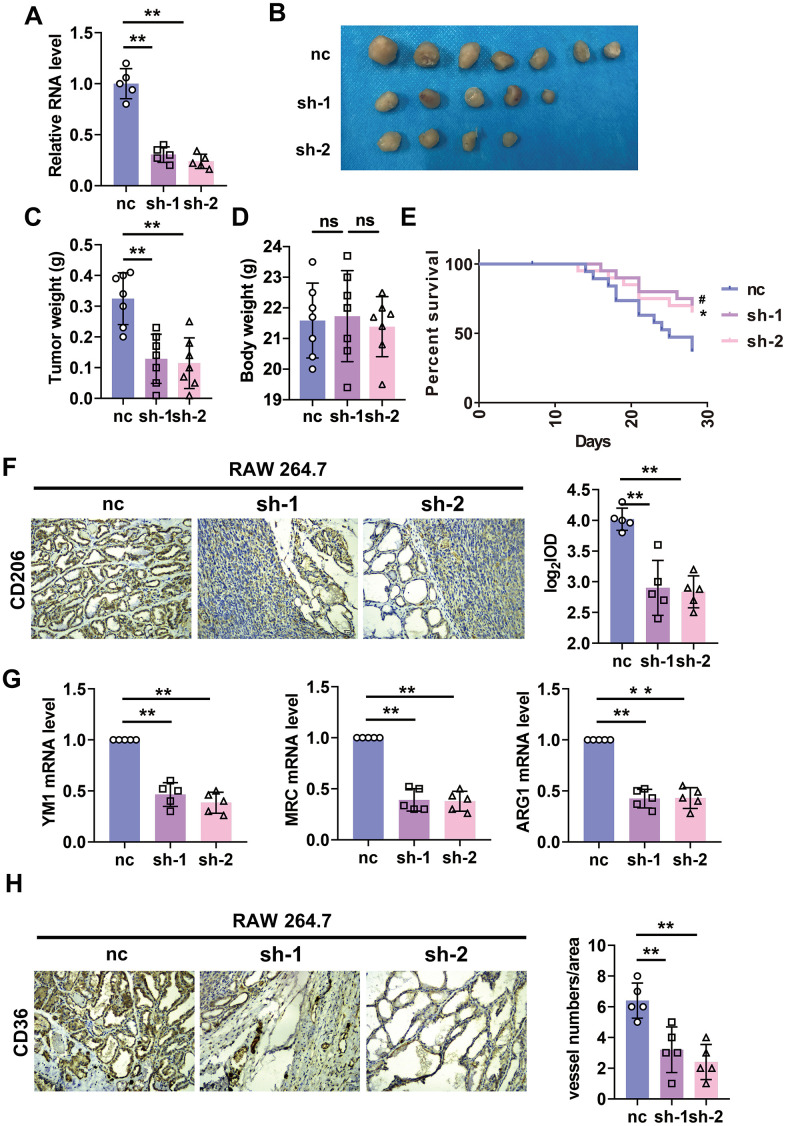
**Silencing of lncRNA-SNHG1 in RAW264.7 cells inhibited tumor growth and angiogenesis.** (**A**) qRT-PCR analysis was performed to measure the RNA level of lncRNA-SNHG1 after transfection of Adv-lncRNA-SNHG1 in RAW264.7 cells. Two-tailed t-test was used for the statistical analysis. n=5 independent cell cultures. The bar indicates the SD values. **P<0.01. (**B**, **C**) The tumors formed in mice at the endpoint of mice study. Two-tailed t-test was used for the statistical analysis. n=7 mice per group. The bar indicates the SD values. **P<0.01. (**D**) Body weight of mice was measured at the endpoint of mice study. Two-tailed t-test was used for the statistical analysis. n=7 mice per group. The bar indicates the SD values. **P<0.01. (**E**) Survival curve was shown by counting dead mice of three groups. n=20 mice per group. ^#^P<0.05 vs. sh-1 group, *P<0.05 vs. sh-2 group. (**F**) IHC staining of CD206 of tumor tissues was performed. Two-tailed t-test was used for the statistical analysis. n=5 mice per group. The bar indicates the SD values. **P<0.01. (**G**) qRT-PCR analysis was performed to measure the RNA level of M2 polarization markers in tumor tissues. n=5 mice per group. The bar indicates the SD values. **P<0.01. (**H**) IHC staining of CD31 of tumor tissues was performed. Two-tailed t-test was used for the statistical analysis. n=5 mice per group. The bar indicates the SD values. **P<0.01.

**Figure 6:**
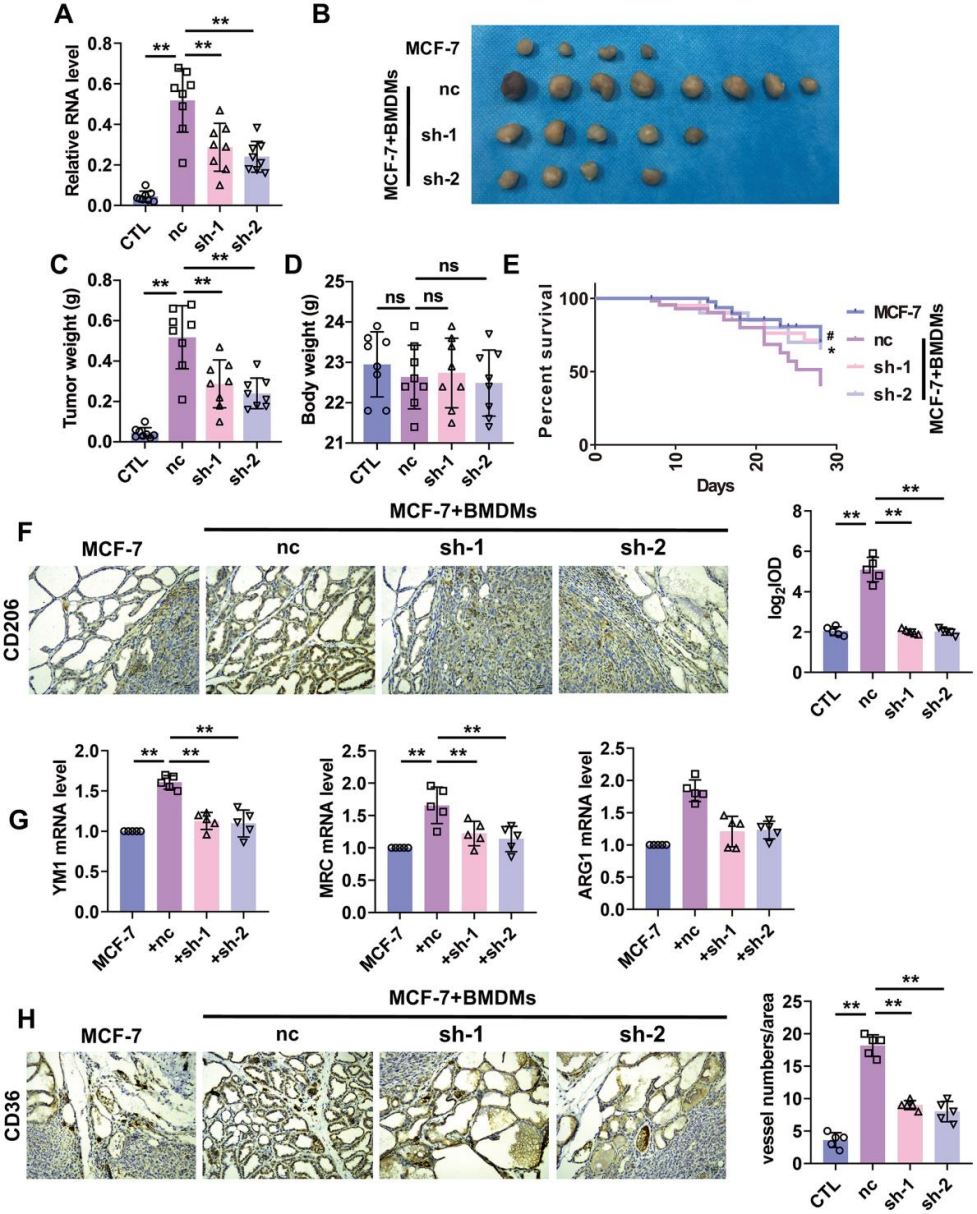
**Silencing of lncRNA-SNHG1 suppressed BMDMs-induced tumor growth and angiogenesis.** (**A**) qRT-PCR analysis was performed to measure the RNA level of lncRNA-SNHG1 after transfection of Adv-lncRNA-SNHG1 in BMDMs. Two-tailed t-test was used for the statistical analysis. n=5 independent cell cultures. The bar indicates the SD values. **P<0.01. (**B**, **C**) The tumors formed in mice at the endpoint of mice study. Two-tailed t-test was used for the statistical analysis. n=7 mice per group. The bar indicates the SD values. **P<0.01. (**D**) Body weight of mice was measured at the endpoint of mice study. Two-tailed t-test was used for the statistical analysis. n=7 mice per group. The bar indicates the SD values. **P<0.01. (**E**) Survival curve was shown by counting dead mice of three groups. n=20 mice per group. ^#^P<0.05, nc group vs. sh-1 group, *P<0.05, nc group vs. sh-2 group. (**F**) IHC staining of CD206 of tumor tissues was performed. Two-tailed t-test was used for the statistical analysis. n=5 mice per group. The bar indicates the SD values. **P<0.01. (**G**) qRT-PCR analysis was performed to measure the RNA level of M2 polarization markers in tumor tissues. n=5 mice per group. The bar indicates the SD values. **P<0.01. (**H**) IHC staining of CD31 of tumor tissues was performed. Two-tailed t-test was used for the statistical analysis. n=5 mice per group. The bar indicates the SD values. **P<0.01.

A mountain of evidence indicated the pro-tumorigenesis and angiogenesis role of M2 macrophage. Here we wanted to explore the role of lncRNA-SNHG1 on macrophage-tumor cell interaction. Firstly, we constructed lentivirus that expressed two shRNA of lncRNA-SNHG1, shRNA1 and shRNA2, and transfected them into RAW264.7 cells. As a result, we got the stable transfected RAW264.7 cell line with lncRNA-SNHG1 knockdown (Figure 5A). Then we implanted the mixture of MCF-7 cells and RAW264.7 cells subcutaneously into the armpits of nude mice for one month. As shown in Figure 5B, the growth of primary tumors showed a difference between control group and lncRNA-SNHG1 silencing group. What’s more, knockdown of lncRNA-SNHG1 in RAW264.7 cells reduced the tumor weight, but not body weight (Figure 5C, 5D). We also implanted cell mixture through tail vein injection into nude mice to evaluate the effect of lncRNA-SNHG1 silencing on survival rate. As shown in Figure 5E, lncRNA-SNHG1 knockdown increased the survival rate of nude mice. IHC staining showed the increased level of CD206^+^ cells in tumor tissue of mice injected with cell mixture that SNHG1 was knockdown (Figure 5F). In addition, M2 polarization markers were also upregulated in tumor tissue of mice injected with cell mixture that SNHG1 was knockdown (Figure 5G). Furthermore, we also measured angiogenesis by CD36 staining. Tumor tissue from the mice injected with silenced lncRNA-SNHG1 cell mixture showed a low level of CD36^+^ cells (Figure 5H). Furthermore, experiments were also repeated by using MCF-7 and BMDMs mixture and showed similar results (Figure 6A–6H). Collectively, these data indicated that lncRNA-SNHG1 silencing in tumor associated macrophage inhibited M2 macrophage polarization and attenuated tumorigenesis and angiogenesis of breast cancer.

## DISCUSSION

Collectively, our data indicated that lncRNA SNHG1 contributed to M2 macrophage polarization via elevating the phosphorylation level of STAT6. Knockdown of lncRNA-SNHG1 attenuated IL4/IL13 induced M2 macrophage polarization. Furthermore, silencing of lncRNA-SNHG1 in macrophages inhibited pro-angiogenesis and tumorigenesis effect of M2-like polarized macrophage.

LncRNA is a kind of noncoding RNA that participates in many physiological and pathological processes, such as cell proliferation and death, development and tumorigenesis. It is well known that lncRNAs play important role in tumorigenesis and metastasis of breast cancer. Zhao et al. found that lncRNA HOTAIR promotes tumorigenesis and metastasis of breast cancer and inhibited cancer cell apoptosis via acting as a ceRNA [23]. PDCD4-AS1 lncRNA contributes to the progression of breast cancer by maintaining the mRNA stability of tumor suppressor gene [24]. Recent work indicated that LINC02273 epigenetically promoted AGR2 transcription and drove breast cancer metastasis [25]. What’s more, Wang et al. reported that lncRNA BM induced breast cancer metastasis by binding with JAK2 [26]. The role and function of lncRNAs in cell-cell interaction of cancer cells and adjacent cells are getting more attention, especially macrophage. Chen et al. found that LNMAT1 promoted recruitment of macrophages and contributed to bladder cancer metastasis [27]. Huang et al. found that lncRNA-MALAT1 increased secretion of FGF2 from tumor associated macrophage and triggered angiogenesis of thyroid cancer [28]. However, the regulation of lncRNAs on macrophage polarization is still poorly understood. Here we found that lncRNA-SNHG1 promoted M2 macrophage polarization. Silencing of lncRNA-SNHG1 by siRNA inhibited M2 macrophage polarization, which was indicated by decreased level of CD206 positive cells. To further determine the role of SNHG1 on M2 macrophage polarization, we detected the effect of lncRNA-SNHG1 silencing on angiogenesis. We found that knockdown of SNHG1 inhibited tube formation *in vitro* and angiogenesis *in vivo*. Our data indicated that lncRNA-SNHG1 localized in cytoplasm of the macrophage. So, we explore the relationship of SNHG1 and M2 macrophage polarization related signaling pathways. Our data demonstrated that lncRNA-SNHG1 promoted M2 macrophage polarization via increased phosphorylation of STAT6 and showed no influence on STAT1 and JAK2.

Breast cancer is the most common tumor in females, the morbidity of which is increasing year by year. The death of breast cancer patients is always due to chemoresistance and cancer metastasis. However, the mechanism of breast cancer development is still poorly understood. As a result, adjustment and treatment of breast cancer is facing a huge challenge. During the development of breast cancer, cancer cells interact with other cells, such as macrophages and endothelial cells, and form the microenvironment that contributes to the growth and metastasis of cancer cells. Tumor associated macrophage (TAM), a kind of immune effector cell, is recruited to cancer tissues to release many kinds of cytokines, chemokines, growth factors, and inflammatory mediators, which play important role in cancer process. The macrophage can comprise up to 50% of the total cell mass in breast cancer. In tumor microenvironment, macrophages under stress can be converted into two subtypes, M1-like and M2-like polarized macrophages. M1-like polarized macrophage is always activated by IFN-Y and LPS, which belong to Th1 cytokines that elaborate the production and secretion of pro-inflammatory cytokines. M1 polarization of macrophages promotes the process of immune response against tumor cells and viral and bacterial infections. However, M2-like polarized macrophage can be activated by IL-4 and IL-13 and plays an anti-inflammatory function. In addition, M2-like polarized macrophage is also involved in the regulation of wound healing, angiogenesis, and tissue remodeling. It has been widely described that M2-like polarized macrophages directly trigger the proliferation and metastasis of breast cancer cells. Here we reported that implantation of mixture of human breast cancer cell line, MCF-7, and macrophages promoted tumor growth and angiogenesis. However, lncRNA-SNHG1 knockdown in macrophages significantly inhibited tumor growth and angiogenesis. Silencing of lncRNA-SNHG1 significantly improved the survival rate of mice after implantation of cell mixture.

Taken together, we indicated the essential role of lncRNA SNHG1 in macrophage polarization and the interaction with breast cancer cells. We will further determine the mechanism that how lncRNA SNHG1 regulates the phosphorylation of STAT6 in our work.

## MATERIALS AND METHODS

### Animals

BALB/c Nude Mice (6-8 weeks old) were obtained from Shanghai Model Organisms (Shanghai, China). All animal experimental procedures are performed according to the protocols of Rizhao People’s Hospital and conformed to the “Guide for the Care and Use of Laboratory Animals” of the National Institute of Health in China.

Mice were separated into three groups randomly, MCF-7 cells+ RAW264.7-control, MCF-7+ RAW264.7-shSNHG1-1 and MCF-7 cells+ RAW264.7-shSNHG1-2. MCF-7 cells (1 X 10^6^) were mixed with RAW264.7 cells (3 X 10^6^) and planted into mice armpits subcutaneously. After 4 weeks, tumor tissues were harvested. Each tumor tissue was weighed and recorded.

For BMDMs experiment, mice were separated into four groups randomly, MCF-7 cells+ BMDMs -control, MCF-7+ BMDMs -shSNHG1-1 and MCF-7 cells+ BMDMs -shSNHG1-2. MCF-7 cells (1 X 10^6^) were mixed with BMDMs (6 X 10^6^) and planted into mice armpits subcutaneously. After 4 weeks, tumor tissues were harvested. Each tumor tissue was weighed and recorded. The animal study was reviewed and approved by the People’s Hospital of Rizhao.

### Cell culture and transfection

MCF-7 cells (human breast cancer cell line) and RAW264.7 cells (murine macrophage cell line) were obtained from Cobioer (Nanjing, China). MCF-7 cells were cultured by RPMI-1640 medium (GIBCO, USA) and RAW264.7 cells were cultured by DMEM (GIBCO, USA). The condition medium contains 10% FBS and 100U/ML penicillin-streptomycin (Sigma-Aldrich, USA). All of the cell lines we used were no more than 20 passages and tested to confirm no mycoplasma contamination.

siRNA1 and 2 of lncRNA-SNHG1 were obtained from RiboBio (Guangzhou, China). Lentivirus that expressed shRNA1 and 2 of lncRNA-SNHG1 with puromycin resistance were obtained from Hanbio (Shanghai, China). Lipo2000 was used to transfect siRNA1 and siRNA2 into RAW264.7 cells cultured with serum-free DMEM. 6h later, the medium was replaced by 10% FBS DMEM. 293A cells were used for virus amplification and the supernatant was harvested and added to RAW264.7 cells and BMDMs to get stable lncRNA-SNHG1 knockout RAW264.7 cell line and lncRNA-SNHG1 knockout BMDMs.

### BMDMs isolation and differentiation

12-week-old C57BL/6 mice were sacrificed by cervical dislocation and sterilized with 75% ethanol. The skin and muscle tissue were removed from the bones of hind legs. Then, the bones were cut from both ends and flushed with medium using a 5-mL syringe. Bone marrow cells were cultured in DMEM (10% FBS) and treated with 50 ng/mL M-CSF (50 ng/mL) for 3d to obtain BMDMs.

### Real-time PCR

Total RNAs were isolated from tissue and cells and reverse transcripted to cDNA using FastKing RT Kit obtained from TianGen (Beijing, China). Then, real-time PCR was performed using SYBR Premix Ex TaqTMa (RR420A, TaKaRa) and following the manufacturer’s instructions.

### Western blot analysis

Total protein was collected using RIPA buffer. Western blotting assay was performed by electrophoresis and blotting system (Bio-Rad, USA) as the manufacturer’s protocol described.

### Transwell assay

Cell suspension (2 × 10^5^ cells/mL) was plated into the upper chamber pre-coated with 1μg/μL Matrigel. The lower chamber was added with 500 μL of medium (10% FBS), and then incubated the chamber at 37° C for 48 h. Then the invading cells were stained by the crystal violet and photographed.

### Tube formation assay

12-well plates were incubated with 500ul Matrigel at 37° C for 2h and planted with HUVEC cells (3 X 10^5^ cells/well). The cells were cultured in condition medium of RAW264.7 cells and BMDMs for 6h. 6 optical fields each well were randomly chosen and photographed.

### FISH assay

Cells were fixed by 4% PFA for 15 min and washed by PBS. 0.4% Triton X-100 was used to permeabilize cells for 15 min Then, cells were incubated with prehybridization buffer. Hybridization buffer needed to be warmed at 37° C. LncRNA, 18s rRNA, and U6 RNA FISH probes were mixed with hybridization buffer and replaced prehybridization buffer. The cells were incubated in a humidified incubator at 37° C overnight. Then 4x SSC (0.6 M NaCl, 0.068 M citric acid, pH 7.0) was used to wash the chamber 3 times in the dark. DAPI was stained for 10 min in the dark. At last, the cells were photographed by confocal microscope (FV1000, Olympus).

### Conditioned medium collection

Macrophage polarization was induced by the treatment of IL4/IL13 (10ng/mL) and LPS/IFNγ (10ng/mL) for 2 days. Then polarized BMDMs and RAW264.7 cells were cultured with serum-free medium for 24h. supernatants of the serum-free medium were collected as conditioned medium (CM) and stored at -80° C.

### Flow cytometry

RAW264.7 cells (5 X 10^4)^ and BMDMs (5 X 10^4)^ were digested and collected. 3% BSA was used to block cells. And then cells were incubated with PE-conjugated CD206, PE-conjugated CD86, or FITC-conjugated F4/80, according to the manufacturers' instructions.

### Immunohistochemistry staining

Tumor tissues were harvested and washed with PBS. 4% PFA was used to fix tumor tissues for 1 day, Tumor tissues were dehydrated by 30% sucrose overnight at 4° C and then frozen in OCT compound. All tissues were cut into 8-mm sections and stained with hematoxylin and eosin (H&E), anti-CD206 (1:500, ab64693, Abcam), or anti-CD31 (1:500, ab28364, Abcam). Secondary antibody (PV-6001, ZSGB-BIO) was incubated at room temperature for 1h. DAB detection kit (ZLI-9018, ZSGB-BIO) was used for color development. At last, hematoxylin counterstaining, hydrochloric acid alcohol differentiation and mounting of the slides were performed.

### Statistical analysis

Statistical analyses were assessed with t-test or one-way ANOVA. All the data are presented as means ± SEM. We used GraphPad Prism 8.0 for all statistics.

## References

[1] Bray F, Ferlay J, Soerjomataram I, Siegel RL, Torre LA, Jemal A. Global cancer statistics 2018: GLOBOCAN estimates of incidence and mortality worldwide for 36 cancers in 185 countries. CA Cancer J Clin. 2018; 68:394–424. 10.3322/caac.21492 30207593

[2] Gong C, Qu S, Lv XB, Liu B, Tan W, Nie Y, Su F, Liu Q, Yao H, Song E. BRMS1L suppresses breast cancer metastasis by inducing epigenetic silence of FZD10. Nat Commun. 2014; 5:5406. 10.1038/ncomms6406 25406648

[3] Junttila MR, de Sauvage FJ. Influence of tumour micro-environment heterogeneity on therapeutic response. Nature. 2013; 501:346–54. 10.1038/nature12626 24048067

[4] Entschladen F, Palm D, Drell TL 4th, Lang K, Zaenker KS. Connecting a tumor to the environment. Curr Pharm Des. 2007; 13:3440–44. 18045197

[5] Shapouri-Moghaddam A, Mohammadian S, Vazini H, Taghadosi M, Esmaeili SA, Mardani F, Seifi B, Mohammadi A, Afshari JT, Sahebkar A. Macrophage plasticity, polarization, and function in health and disease. J Cell Physiol. 2018; 233:6425–40. 10.1002/jcp.26429 29319160 PMC13482560

[6] Wang N, Liang H, Zen K. Molecular mechanisms that influence the macrophage m1-m2 polarization balance. Front Immunol. 2014; 5:614. 10.3389/fimmu.2014.00614 25506346 PMC4246889

[7] DeNardo DG, Barreto JB, Andreu P, Vasquez L, Tawfik D, Kolhatkar N, Coussens LM. CD4(+) T cells regulate pulmonary metastasis of mammary carcinomas by enhancing protumor properties of macrophages. Cancer Cell. 2009; 16:91–102. 10.1016/j.ccr.2009.06.018 19647220 PMC2778576

[8] Shiao SL, Ruffell B, DeNardo DG, Faddegon BA, Park CC, Coussens LM. TH2-Polarized CD4(+) T Cells and Macrophages Limit Efficacy of Radiotherapy. Cancer Immunol Res. 2015; 3:518–25. 10.1158/2326-6066.CIR-14-0232 25716473 PMC4420686

[9] Shieh YS, Hung YJ, Hsieh CB, Chen JS, Chou KC, Liu SY. Tumor-associated macrophage correlated with angiogenesis and progression of mucoepidermoid carcinoma of salivary glands. Ann Surg Oncol. 2009; 16:751–60. 10.1245/s10434-008-0259-6 19116756

[10] Kale S, Raja R, Thorat D, Soundararajan G, Patil TV, Kundu GC. Osteopontin signaling upregulates cyclooxygenase-2 expression in tumor-associated macrophages leading to enhanced angiogenesis and melanoma growth via α9β1 integrin. Oncogene. 2014; 33:2295–306. 10.1038/onc.2013.184 23728342

[11] Jarroux J, Morillon A, Pinskaya M. History, Discovery, and Classification of lncRNAs. Adv Exp Med Biol. 2017; 1008:1–46. 10.1007/978-981-10-5203-3_1 28815535

[12] Ballantyne MD, Pinel K, Dakin R, Vesey AT, Diver L, Mackenzie R, Garcia R, Welsh P, Sattar N, Hamilton G, Joshi N, Dweck MR, Miano JM, et al. Smooth Muscle Enriched Long Noncoding RNA (SMILR) Regulates Cell Proliferation. Circulation. 2016; 133:2050–65. 10.1161/CIRCULATIONAHA.115.021019 27052414 PMC4872641

[13] Fatica A, Bozzoni I. Long non-coding RNAs: new players in cell differentiation and development. Nat Rev Genet. 2014; 15:7–21. 10.1038/nrg3606 24296535

[14] Schmitt AM, Chang HY. Long Noncoding RNAs in Cancer Pathways. Cancer Cell. 2016; 29:452–63. 10.1016/j.ccell.2016.03.010 27070700 PMC4831138

[15] Kopp F, Mendell JT. Functional Classification and Experimental Dissection of Long Noncoding RNAs. Cell. 2018; 172:393–407. 10.1016/j.cell.2018.01.011 29373828 PMC5978744

[16] Huarte M. The emerging role of lncRNAs in cancer. Nat Med. 2015; 21:1253–61. 10.1038/nm.3981 26540387

[17] Bhan A, Soleimani M, Mandal SS. Long Noncoding RNA and Cancer: A New Paradigm. Cancer Res. 2017; 77:3965–81. 10.1158/0008-5472.CAN-16-2634 28701486 PMC8330958

[18] Li Z, Lu Q, Zhu D, Han Y, Zhou X, Ren T. Lnc-SNHG1 may promote the progression of non-small cell lung cancer by acting as a sponge of miR-497. Biochem Biophys Res Commun. 2018; 506:632–40. 10.1016/j.bbrc.2018.10.086 30454699

[19] Li HM, Yu YK, Liu Q, Wei XF, Zhang J, Zhang RX, Sun HB, Wang ZF, Xing WQ, Li Y. LncRNA SNHG1 Regulates the Progression of Esophageal Squamous Cell Cancer by the miR-204/HOXC8 Axis. Onco Targets Ther. 2020; 13:757–67. 10.2147/OTT.S224550 32158227 PMC6986417

[20] Li SJ, Wang L, Sun ZX, Sun SJ, Gao J, Ma RL. LncRNA SNHG1 promotes liver cancer development through inhibiting p53 expression via binding to DNMT1. Eur Rev Med Pharmacol Sci. 2019; 23:2768–76. 10.26355/eurrev_201904_17550 31002127

[21] Cui Y, Zhang F, Zhu C, Geng L, Tian T, Liu H. Upregulated lncRNA SNHG1 contributes to progression of non-small cell lung cancer through inhibition of miR-101-3p and activation of Wnt/β-catenin signaling pathway. Oncotarget. 2017; 8:17785–94. 10.18632/oncotarget.14854 28147312 PMC5392286

[22] Lu Q, Shan S, Li Y, Zhu D, Jin W, Ren T. Long noncoding RNA SNHG1 promotes non-small cell lung cancer progression by up-regulating MTDH via sponging miR-145-5p. FASEB J. 2018; 32:3957–67. 10.1096/fj.201701237RR 29466052

[23] Zhao W, Geng D, Li S, Chen Z, Sun M. LncRNA HOTAIR influences cell growth, migration, invasion, and apoptosis via the miR-20a-5p/HMGA2 axis in breast cancer. Cancer Med. 2018; 7:842–55. 10.1002/cam4.1353 29473328 PMC5852357

[24] Jadaliha M, Gholamalamdari O, Tang W, Zhang Y, Petracovici A, Hao Q, Tariq A, Kim TG, Holton SE, Singh DK, Li XL, Freier SM, Ambs S, et al. A natural antisense lncRNA controls breast cancer progression by promoting tumor suppressor gene mRNA stability. PLoS Genet. 2018; 14:e1007802. 10.1371/journal.pgen.1007802 30496290 PMC6289468

[25] Xiu B, Chi Y, Liu L, Chi W, Zhang Q, Chen J, Guo R, Si J, Li L, Xue J, Shao ZM, Wu ZH, Huang S, Wu J. LINC02273 drives breast cancer metastasis by epigenetically increasing AGR2 transcription. Mol Cancer. 2019; 18:187. 10.1186/s12943-019-1115-y 31856843 PMC6921600

[26] Wang S, Liang K, Hu Q, Li P, Song J, Yang Y, Yao J, Mangala LS, Li C, Yang W, Park PK, Hawke DH, Zhou J, et al. JAK2-binding long noncoding RNA promotes breast cancer brain metastasis. J Clin Invest. 2017; 127:4498–515. 10.1172/JCI91553 29130936 PMC5707156

[27] Chen C, He W, Huang J, Wang B, Li H, Cai Q, Su F, Bi J, Liu H, Zhang B, Jiang N, Zhong G, Zhao Y, et al. LNMAT1 promotes lymphatic metastasis of bladder cancer via CCL2 dependent macrophage recruitment. Nat Commun. 2018; 9:3826. 10.1038/s41467-018-06152-x 30237493 PMC6148066

[28] Huang JK, Ma L, Song WH, Lu BY, Huang YB, Dong HM, Ma XK, Zhu ZZ, Zhou R. LncRNA-MALAT1 Promotes Angiogenesis of Thyroid Cancer by Modulating Tumor-Associated Macrophage FGF2 Protein Secretion. J Cell Biochem. 2017; 118:4821–30. 10.1002/jcb.26153 28543663

